# Is Knowledge Regarding Tuberculosis Associated with Stigmatising and Discriminating Attitudes of General Population towards Tuberculosis Patients? Findings from a Community Based Survey in 30 Districts of India

**DOI:** 10.1371/journal.pone.0147274

**Published:** 2016-02-01

**Authors:** Karuna D. Sagili, Srinath Satyanarayana, Sarabjit S. Chadha

**Affiliations:** International Union against Tuberculosis and Lung Disease, South-East Asia Regional Office, New Delhi, India; Public Health Research Institute at RBHS, UNITED STATES

## Abstract

**Background:**

Stigmatising and discriminating attitudes may discourage tuberculosis (TB) patients from actively seeking medical care, hide their disease status, and discontinue treatment. It is expected that appropriate knowledge regarding TB should remove stigmatising and discriminating attitudes. In this study we assessed the prevalence of stigmatising and discriminating attitudes towards TB patients among general population and their association with knowledge regarding TB.

**Method:**

A cross-sectional knowledge, attitude and practice survey was conducted in 30 districts of India in January-March 2011. A total of 4562 respondents from general population were interviewed using semi-structured questionnaires which contained items to measure stigma, discrimination and knowledge on TB.

**Result:**

Of the 4562 interviewed, 3823 were eligible for the current analysis. Of these, 73% (95% CI 71.4–74.2) had stigmatising and 98% (95% CI 97.4–98.3) had discriminating attitude towards TB patients. Only 17% (95% CI 15.6–18.0) of the respondents had appropriate knowledge regarding TB with even lower levels observed amongst females, rural areas and respondents from low income groups. Surprisingly stigmatising (adjusted OR 1.31 (0.78–2.18) and discriminating (adjusted OR 0.79 (0.43–1.44) attitudes were independent of knowledge regarding TB.

**Conclusion:**

Stigmatising and discriminating attitudes towards TB patients remain high among the general population in India. Since these attitudes were independent of the knowledge regarding TB, it is possible that the current disseminated knowledge regarding TB which is mainly from a medical perspective may not be adequately addressing the factors that lead to stigma and discrimination towards TB patients. Therefore, there is an urgent need to review the messages and strategies currently used for disseminating knowledge regarding TB among general population and revise them appropriately. The disseminated knowledge should include medical, psycho-social and economic aspects of TB that not only informs people about medical aspects of TB disease, but also removes stigma and discrimination.

## Introduction

Tuberculosis (TB) is considered as a social disease, with many socio-cultural factors contributing to the disease burden [1].With an estimated 2.6 million incident TB cases in 2013 [2], the Revised National TB Control Program (RNTCP) of India majorly relies on passive case finding. In this context, an effective alliance with patients and their communities is essential for timely diagnosis and favourable treatment outcomes. However, this alliance may be affected by stigma and discrimination, resulting in patients with TB symptoms (presumptive TB patients) shying away from getting tested and not taking treatment if diagnosed with TB. Recent studies on social and behavioural aspects related to TB have established the presence of stigma towards TB patients [3,4,5].Studies from India, Bangladesh, Malawi and Columbia showed that due to stigma associated with TB, an individual with TB-like symptoms may avoid or delay seeking care, conceal disease status or discontinue treatment [3].

‘Stigma’ in general, is defined [6] as a process within a given cultural setting that discredits/devalues an individual based on an undesired attribute. Stigma is a powerful means of social control applied by labelling, marginalising, stereotyping, excluding and exercising power over individuals who display certain traits/affected by perceived unacceptable attributes including health related issues. It also influences the way an individual perceives himself /herself [7]. While “stigma” is an attitude or belief, “discrimination” is behaviour because of those attitudes or beliefs. Discrimination occurs when individuals with stigmatising attitudes deny/prevent others of their rights and life opportunities by excluding or marginalizing them [8].

TB related stigma negatively influences lives of TB patients and their families making them vulnerable to poor quality of life. Stigma and discrimination co-exist at family and societal levels taking into account various regional, cultural and economical aspects. A baseline KAP study conducted by RNTCP [5] in 2003 showed that stigma and discrimination towards TB patients exists along with gender discrimination towards female patients. A study in Tamil Nadu [9] showed that stigma and discrimination continues to exist though some improvements were seen in certain areas. A study conducted in Delhi [10] observed that about 60% TB patients hide their disease from other members of the community due to fear of stigmatisation; this was more among middle and upper class people.

It is hypothesized that TB patients are stigmatised and discriminated due to incorrect knowledge or awareness about TB and myths about TB [11, 12].Having appropriate or adequate knowledge regarding TB (KTB) disease is expected to also address other psycho-social effects of TB, positively changing any existing stigmatising or discriminating attitudes (SDA). However, the key TB related knowledge facts currently disseminated in various awareness programs focus on the microbiological cause of TB, its symptoms, treatment and curability with a full course of anti-TB medications[13, 14]. Hence in the present paper we assessed the prevalence of SDA towards TB patients among general population and their association with KTB, specifically its symptoms, transmission and treatment among general population.

## Materials and Methods

### Study Setting

This study was conducted as part of Project Axshya [an Advocacy, Communication and Social Mobilisation (ACSM) project], funded by The Global Fund in India. Project Axshya aims to increase civil society’s support to the national TB program and to engage communities and community based care providers in 374 out of 650 districts across 21 of the 35 states and union territories in the country.

### Study Design, sample size, sampling and study population

A cross-sectional community based knowledge, attitude and practice (KAP) survey about TB was conducted in a representative sample of 30 districts in India in January-March 2011.These 30 districts were selected from 374 districts by a stratified clustered sampling method. Initially, the 374 districts were stratified by zone as per RNTCP (north, west, east and south) and the number of districts required in each zone was estimated based on distribution of the districts in different zones. The number of districts in each zone was select by population proportionate to size sampling method. More details of selection of the districts and sampling have been discussed in detail elsewhere [15].Within each district, 10 primary sampling units (PSU), each of ~250 households each was selected. Within each PSU, all household members aged ≥18 years were line listed and from this line list 15 respondents were identified in a systematic random manner.

### Study investigators, data collection, study instruments and study variables

The Union, South East Asia office conducted this study with the assistance of GfK MODE, a social research agency. All respondents were informed about the study and after obtaining consent, were administered with a closed-ended survey tool by trained investigators. The survey instruments were pretested, translated, and piloted after the translations.Only those who gave consent were included in the study.

The key study variables collected included socio-demographic information like age, sex, education, income; knowledge about TB, its symptoms, route of transmission, diagnosis, treatment, duration of treatment, risk factors and statements to capture stigmatising and discriminating attitudes.

#### Exclusion criteria

Adults (18years and above) identified during the survey were invited to participate and those who gave consent were included. Those households with a TB patient who is either on treatment at the time of survey or one year before the survey were excluded from the study.

A total of 4562 individuals from general population were interviewed. Respondents who heard about TB or knew about TB were included in this study.

### Operational definitions

#### Stigmatising attitude

An attitude is an ‘outlook’ or ‘a belief’ or ‘a mind-set’ that has the potential to influence our actions. Respondents were expected to either agree or disagree to a set of seven statements (Table 1). If someone agreed for 3 or more statements, we have operationally considered that the person has a stigmatising attitude towards TB patients.

**Table 1:** Statements as part of the KAP survey tool to capture stigmatising and discriminating attitudes.

**Stigmatising Attitudes**	**Now I Will Make Some Statements About People Suffering From Tb. Please Let Me Know How Much You Agree To These?**	Strongly Agree*	Somewhat Agree*	**Disagree**
1	A family with TB patient should not be allowed to participate in any social function			
2	Married female TB patient should be sent off to her parent’s house			
3	Children with TB should not be allowed to go to school			
4	Children of parents suffering from TB should not be allowed to go to school			
5	Daily wage Laborer, suffering from TB should not be allowed to work			
6	TB patient are threat to community			
7	TB patients should be left isolated in the community			
**Discriminating Attitudes**	**Which of the following you would agree to do?**	**Yes**	**No**	** DK/CS
1	Share a meal with person you know had TB			
2	If you suspect one of the female member is suffering from TB, would you take her to hospital			
3	Marry your daughter to a boy knowing had a TB			
4	Isolate your family member having TB in the house			
5	Marry your son to a girl who you know had TB			
6	Send your daughter in law to parent’s house if she had TB in order to protect other family members from TB			

* Strongly agree and somewhat agree have been considered as affirmatives for stigmatising attitudes

** Don’t know/Can’t say

#### Discriminating attitude

A negative action towards an individual based on his/her health or social status is considered as discriminating attitude. A set of six hypothetical statements (Table 1) were asked to the respondents for whom they were expected to respond ‘yes’ or ‘no’. If someone said ‘yes’ to 3 or more statements, we have operationally considered that the person has a discriminating attitude towards TB patients.

#### Knowledge regarding TB

Appropriate knowledge–Individuals were considered to be having appropriate knowledge regarding TB if they knew all of the following a) cough of 2 weeks or more is the key symptom of TB, b) TB is a curable disease, c) had heard of DOTS (Direct Observed Treatment, Short course) programme implemented by the Government of India and d) knew that TB is transmitted through air from an infected person when he/she coughs or sneezes.

Partial knowledge–Those individuals who knew at least one of the above mentioned indicators were considered to have partial knowledge regarding TB.

### Data entry and analysis

Data collected were entered into pre-designed formats in Fox Pro (Version 2.6) and were cross-verified for consistency. The data was analysed using EpiData Analysis software (Version 3.1.80) and Stata (Version 12). Dataset is made available as per journal requirements (S1 Dataset). Stigmatising and discriminating attitudes were quantified based on the responses given to the statements described above. Statistical associations between different variables in the sub-groups of the study population were done using Chi square test. A p value of <0.05 was considered statistically significant. Multivariate logistic regression was carried out to obtain the odds ratio and adjusted odds ratio for the independent association between knowledge, stigma and discrimination with socio-demographic factors such as age, sex, education levels, income levels, geographic location after accounting for clustering of at the district level.

### Ethics Considerations

The study protocol was approved by the Ethics Advisory Group of The Union. In addition, as this survey was an approved activity under the Global Fund grant, Central TB Division, Ministry of Health and Family Welfare, Government of India provided consent to the various aspects of the survey. Prior to conducting the survey, permission was also sought from the community heads/representatives of the primary sampling units in each district. Informed written consent was also sought from the heads of households and individual respondents.

## Results

A total of 3823 respondents who mentioned that they heard (i.e. those who had an idea of TB/what TB was) about TB were included in the study.More than half of the respondents were male (53%),were literate (65%), from rural settlements (72%)and from lower income group (60%).

### Knowledge about TB (KTB)

Only 17% (N = 641, 95% CI 15.6–18.0) had appropriate KTB (as defined in methods) (Table 2). Various demographic factors like sex, type of settlement, literacy and income were associated with KTB. After adjusting for these factors, it was observed that respondents between the ages 35years-54years had less chance of having appropriate KTB when compared to respondents in 18–24 year age-group (adjusted OR 35–44 years 0.64 (0.48–0.87); adjusted OR 45-54years 0.55 (0.41–0.73, Table 3). Respondents living in urban settlements, from the west zone, and from middle-high income groups have twice higher chance of having appropriate KTB when compared to respondents living in rural areas, respondents from north zone and respondents from low-income groups respectively (Table 3).

**Table 2:** Proportions of respondents with knowledge regarding TB, stigmatising and discriminating attitudes towards TB patients among general population from 30 districts in India in 2011.

	Demographic characteristics	Knowledge regarding TB (N = 3823)	Stigmatising attitude (N = 3793)	Discriminating attitude (N = 3571)
		Appropriate	Yes	Yes
**Overall**		641 (17%) [95% CI 15.6–18.0]	2761 (73%) [95% CI 71.4–74.2]	3497 (98%) [95% CI 97.4–98.3]
**By Knowledge**	**Appropriate (N = 641)**	NA	457 (71%)	553 (98%)
	**Partial (N = 3182)**	NA	2304 (73%)	2944 (98%)
**Sex**	**Male (N = 2017)**	368 (18%)	1458 (73%)	1840 (98%)
	**Female (N = 1806)**	273 (15%)	1303 (73%)	1657 (98%)
**Type of settlement**	**Rural (N = 2759)**	318 (12%)	2077 (76%)	2566 (98%)
	**Urban (N = 1064)**	323 (30%)	684 (64%)	931 (98%)
**Literacy**	**Illiterate (N = 1336)**	95 (7%)	940 (71%)	1256 (99%)
	**Literate (N = 2487)**	546 (22%)	1821 (74%)	2241 (98%)
Income groups*	**Low income (N = 2292) (upto $63 per month)**	257 (11%)	1614 (71%)	2160 (99%)
	**Middle income (N = 1225) ($63-$159 per month)**	280 (23%)	892 (73%)	1060 (97%)
	**High income (N = 254) (>$159 per month)**	100 (39%)	222 (87%)	230 (98%)
**Zone**	**East (N = 1055)**	210 (20%)	879 (83%)	1021 (99%)
	**West (N = 935)**	311 (33%)	361 (40%)	857 (98%)
	**North (N = 1021)**	170 (17%)	853 (84%)	972 (98%)
	**South (N = 812)**	128 (16%)	668 (82%)	647 (95%)

* In US $ @ INR 63 = $1 (accessed on 3^rd^ July 2015)

**Table 3:** Factors associated with stigmatising and discriminating attitudes towards TB patients by the general population across 30 districts in India in 2011.

	Knowledge of TB		Stigmatising attitude		Discriminating attitude	
	OR (95% CI)	Adjusted OR* (95% CI)	OR (95% CI)	Adjusted OR** (95% CI)	OR (95% CI)	Adjusted OR** (95% CI)
**Age group**						
18–24	Referent	Referent	Referent	Referent	Referent	Referent
25–34	0.88 (0.72–1.07)	0.89 (0.71–1.12)	0.87 (0.66–1.14)	0.89 (0.68–1.18)	0.71 (0.21–2.41)	0.67 (0.19–2.41)
35–44	0.57 (0.43–0.75)	0.64 (0.48–0.87)	0.84 (0.67–1.05)	0.92 (0.70–1.20)	0.68 (0.21–2.26)	0.63 (0.17–2.28)
45–54	0.51 (0.37–0.69)	0.55 (0.41–0.73)	0.82 (0.62–1.09)	0.82 (0.62–1.10)	0.55 (0.21–1.45)	0.56 (0.17–1.78)
55–65	0.41 (0.15–1.13)	0.50 (0.21–1.23)	1.02 (0.49–2.13)	0.88 (0.43–1.79)	0.31 (0.15–0.67)	0.44 (0.15–1.26)
**Sex**						
Male	Referent	Referent	Referent	Referent	Referent	Referent
Female	0.79 (0.64–0.99)	0.85 (0.68–1.05)	0.99 (0.80–1.22)	0.96 (0.74–1.23)	1.39 (0.98–1.98)	1.32 (0.92–1.90)
**Type of settlement**						
Rural	Referent	Referent	Referent	Ref	Referent	Referent
Urban	3.34 (1.85–6.04)	2.20 (1.28–3.77)	0.57 (0.21–1.51)	0.72 (0.37–1.39)	0.92 (0.30–2.77)	1.15 (0.40–3.31)
**Zone**						
North	Referent	Referent	Referent	Referent	Referent	Referent
South	1.16 (0.38–3.47)	0.79 (0.32–2.02)	0.87 (0.19–3.99)	0.88 (0.19–3.99)	0.33 (0.8–1.43)	0.40 (0.99–1.65)
East	1.30 (0.36–4.71)	1.20 (0.53–2.72)	0.94 (0.36–2.47)	0.97 (0.38–2.48)	2.97 (0.96–9.20)	3.02 (0.94–9.76)
West	2.34 (1.59–3.45)	2.08 (1.27–3.38)	0.12 (0.04–0.36)	0.13 (0.04–0.39)	0.88 (0.18–4.19)	0.88 (0.20–3.85)
**Education**						
Illiterate	Referent	Referent	Referent	Referent	Referent	Referent
Literate	3.67 (2.17–6.21)	2.26 (1.46–3.49)	1.14 (0.73–1.79)	1.17 (0.86–1.61)	0.57 (0.31–1.06)	0.90 (0.42–1.93)
**Income group**						
Low	Referent	Referent	Referent	Referent	Referent	Referent
Middle	2.34 (1.46–3.75)	1.86 (1.26–2.72)	1.11 (0.52–2.37)	0.98 (0.63–1.55)	0.41 (0.19–0.89)	0.51 (0.24–1.09)
High	5.14 (2.55–10.36)	3.94 (2.47–6.29)	2.82 (1.50–5.28)	2.25 (1.29–3.91)	0.85 (0.31–2.37)	0.94 (0.24–3.76)
**Knowledge of TB**						
Appropriate	NA	NA	Referent	Referent	Referent	Referent
Partial	NA	NA	0.92 (0.49–1.73)	1.31 (0.78–2.18)	0.81 (0.38–1.70)	0.79 (0.43–1.44)

* Adjusted for age, sex, education, geographical zone and income group

** Adjusted for age, sex, education, geographical zone, income group and knowledge regarding TB

### Stigmatising and discriminating attitudes (SDA)

Overall, stigmatising attitude was present in 73% (N = 2761, 95% CI 71.4–74.2) and discriminating attitude in 98% (N = 3497, 95% CI 97.4–98.3) of the respondents (Table 2). Individual responses to statements of stigma and discrimination are presented in Table 4. Associations with socio-demographic factors like age, sex, literacy and income were studied and adjusted odds ratios were calculated. It was observed that respondents from high income group had twice higher chance to have stigmatising attitudes when compared to respondents from low income group (adjusted odds ratio 2.25 (1.29–3.91, Table 3).

**Table 4:** Responses to the statements used to quantify stigmatising (those who agreed to the statements) and discriminating attitudes (those who said ‘No’ to the statements) (N = 3823) towards TB patients among general population from 30 districts in India in 2011.

	**Statements to capture stigmatising attitude**	**N (%)**	**95% CI**
1	A family with TB patient should not be allowed to participate in any social function	1392 (36)	34.9–37.9
2	Married female TB patient should be sent off to her parent’s house	775 (20)	19.0–21.6
3	Children with TB should not be allowed to go to school	1712 (45)	43.2–46.4
4	Children of parents suffering from TB should not be allowed to go to school	1408 (37)	35.3–38.4
5	Daily wage Labourer, suffering from TB should not be allowed to work	1956 (51)	49.6–52.7
6	TB patient are threat to community	2366 (62)	60.3–63.4
7	TB patients should be left isolated in the community	1412 (37)	35.4–38.5
	**Statements to capture discriminating attitude**		
1	Will you share a meal with person you know had TB	3102 (81)	79.9–82.4
2	If you suspect one of the female member of your family is suffering from TB, would you take her to hospital	313 (8)	7.4–9.1
3	Will you marry your daughter to a boy knowing had a TB	2851 (75)	73.2–75.9
4	Will you isolate your family member having TB in the house	1032 (27)	25.6–28.4
5	Will you marry your son to a girl who you know had TB	2956 (77)	76.0–78.6
6	Will you send your daughter in law to her parent’s house if she had TB in order to protect other family members from TB	398 (10)	9.5–11.4

### Association of KTB and SDA

Tables 5 and 6 summarize the proportions of respondents having SDA among the respondents with individual TB knowledge indicators. Three-fourths of respondents who knew that TB transmits through air when an infected person coughs or sneezes had stigmatising attitudes (74%, OR = 1.20, 195% CI 1.039–1.389) when compared to people who did not know about this. However, all most all without this knowledge had discriminating attitudes (99%, OR = 0.489 (95% CI 0.289–0.829). At the same time three-fourths of respondents knowing that TB is curable had stigmatising attitudes (74%, OR = 1.504 (95% CI 1.225–1.847) when compared to those who did not know this.

**Table 5:** Proportions of respondents who were aware of individual TB knowledge indicators among those who had stigmatising attitudes towards TB patients from 30 districts in India in 2011.

Knowledge indicators		Stigmatising attitude N = 3793	Odds Ratio (OR) (95% Confidence interval) and p value
		N (%**)	
**Know that cough of >2 weeks could be TB**	Yes (n = 2833)	2041 (72)	OR = 0.86 (0.73–1.01) p = 0.075
	No (n = 960)	720 (75)	
**Know that TB is curable**	Yes (n = 3324)	2455 (74)	OR = 1.50 (1.22–1.85) p = 0.000*
	No (n = 469)	306 (65)	
**Know that TB is transmitted through air when an infected person coughs or sneezes**	Yes (n = 2280)	1693 (74)	OR = 1.20 (1.04–1.39) p = 0.013*
	No (n = 1513)	1068 (71)	
**Heard about DOTS**	Yes (n = 1053)	729 (69)	OR = 0.78 (0.67–0.92) p = 0.002*
	No (n = 2740)	2032 (74)	

*Significant

** Row percentages

**Table 6:** Proportions of respondents who were aware of individual TB knowledge indicators among those who had discriminating attitudes towards TB patients from 30 districts in India in 2011.

Knowledge indicators		Discriminating attitude (N = 3497)	Odds Ratio (OR) (95% Confidence interval) and p value
		N (%**)	
**Know that cough of >2 weeks could be TB**	Yes (n = 2657)	2601 (98)	OR = 0.93 (0. 54–1.59) p = 0.800
	No (n = 914)	896 (98)	
**Know that TB is curable**	Yes (n = 3114)	3045 (98)	OR = 0. 49 (0 .19–1.22) p = 0.116
	No (n = 457)	452 (98)	
**Know that TB is transmitted through air when an infected person coughs or sneezes**	Yes (n = 2106)	2051 (97)	OR = 0 .49 (0.29–0.89) p = 0.007*
	No (n = 1465)	1446 (99)	
**Heard about DOTS**	Yes (n = 960)	939 (98)	OR = 0 .93 (0.55–1.54) p = 0.77
	No (n = 2611)	2558 (98)	

*Significant

** Row percentages

However, after adjusting for the socio-demographic factors it was observed that KTB had no association with SDA.

## Discussion

Stigmatising and discriminating attitudes towards TB patients were present among three-fourths of the general population in India. Previous studies documented the presence of stigma towards TB patients at family and societal or community level and gender bias [8,9, 10, 11, 16, 17, 18, 19]. A recent systematic review on stigma and tuberculosis concluded that cultural and economical variations related to gender, marriage, employment play an important role in stigmatisation of TB patients [12]. A recent study on perception of community members towards leprosy and TB patients in Thailand documented negative attitudes towards those affected with leprosy and TB [20], recommending tailor-made interventions to address stigma.

KTB was heterogeneously distributed across gender, settlements, literacy groups and social strata. The observations from the study showed association of appropriate KTB with urban settlements and from higher income group. Even among those who had appropriate knowledge, SDA existed. For example, higher proportion of respondents from high income groups not only had appropriate knowledge but also stigmatising attitudes when compared with middle or low income groups. West zone districts from the study (mainly from Maharashtra) stand out with highest proportions of appropriate knowledge and lowest proportions of stigmatising attitudes. The reasons for the same need to be further investigated.

Respondents who were aware that TB is curable and is transmitted through air had high stigmatising attitudes, suggesting that mere knowledge about curability or transmission route may be insufficient to remove such attitudes. Overall, knowledge regarding symptoms, transmission and curability of TB does not seem to eliminate stigma and discrimination around TB. This could be attributed to the fact that the current disseminated KTB is most often from a microbiological and medical perspective such as symptoms, transmission, treatment and curability. For instance, the most recent documents and reports about TB highlight the cause, symptoms, treatment and curability [14, 21, 22]. While the communication strategy of RNTCP emphasises on addressing stigma and discrimination, most of the IEC materials do not include relevant messages. The RNTCP’s communication strategy framework also suggests KTB to be contextualised and not be limited to medical or biological information [23]. It clarifies that information alone cannot de-stigmatise TB, the underlying fears of infection and death need to be addressed. The operational handbook on ACSM developed by RNTCP and USAID in 2013 suggests five goals of ACSM; combating stigma and discrimination being one of them [24]. It also suggests TB curability as a key message to combat stigma. However in our study we observed that individuals who knew TB was curable had stigmatising attitudes significantly higher than those who did not know this.

Though it is documented that TB patient’s psycho-social status is affected by stigma and discrimination [9], not much is done to address this. The social aspects and myths about TB are not dealt in a systematic way. The underlying personal or familial fears such as fear of infection and death due to TB, fear of loss of livelihood and social life, fear of losing social status, fear of rejection in family and society including reduced marriage prospects and fatalistic beliefs are either unaddressed or under-addressed [8]. Messages addressing these fears need to be communicated across all age groups, regions and strata of society. Association of TB with poverty, low caste and HIV-AIDS have also been documented [10]. In the Indian context, the associations with low caste and low class could play an important role in stigmatisation or discrimination as these are not usually addressed in the awareness or educational programs on TB. For example, in some Indian villages people from scheduled castes live in separate yet identifiable clusters (called harijan basti). They are often marginalised at various levels and are vulnerable for TB disease due to risk factors like poverty, malnutrition or smoking and not because of their caste. So when the health programs focus on these clusters for TB control, the possibility of other communities around linking their caste to TB disease is very much possible. Hence, cultural factors, personal/familial fears and societal status could be sustaining factors of stigma and discrimination towards TB patients in India.

Currently the key knowledge aspects that are being disseminated in various awareness programs, and those included in the survey, deal with symptoms, transmission, treatment and curability. These do not include any social aspects. It is possible that this current disseminated KTB may be inadequate to address the factors that lead to SDA towards TB patients. Therefore, there is an urgent need to integrate social aspects into our TB control program. The current disseminated KTB needs to be reviewed and revised appropriately. The information should include medical, psycho-social and economical aspects addressing the underlying fears that contribute to the TB disease burden. Addressing the underlying fears about TB could remove the barriers of stigma and discrimination and could accelerate end TB strategy in India. Future research could focus on understanding the root causes of stigma and discrimination and pilot testing tailor-made strategies.

### Limitations

Health related stigma can be measured or assessed using five different approaches [16] which includes assessment of attitudes and practices. The findings described in this study are from a large cross-sectional knowledge, attitude and practice survey which used semi-quantitative (close-ended) tools. While the large scale is strength of the study, public attitudes may or may not result in actual experiences; hence the findings need to be further understood using qualitative methods.

## Supporting Information

S1 Dataset Project Axshya KAP survey 2011. (XLS)
